# The relationship between moral distress and occupational burnout among ICU nurses: social support and psychological resilience as mediating variables

**DOI:** 10.3389/fpubh.2025.1743774

**Published:** 2026-01-12

**Authors:** Fengzhi Chai, Wei Hu, Di Xu, Yunfan Ji, Yuhong Wang, Caiyue Xu, Xia Li

**Affiliations:** 1 First Affiliated Hospital of Jinzhou Medical University, Jinzhou, Liaoning, China; 2 School of Nursing, Jinzhou Medical University, Jinzhou, Liaoning, China

**Keywords:** chain mediation, intensive care unit, moral distress, occupational burnout, psychological resilience, social support

## Abstract

**Background:**

ICU nurses are prone to occupational burnout due to high workloads and emotional exhaustion. Moral distress exacerbates this issue, though the precise mechanisms remain unclear.

**Objective:**

This study aims to examine the mediating role of social support and psychological resilience in the relationship between moral distress and occupational burnout among ICU nurses.

**Methods:**

A cross-sectional survey was conducted from October to November 2024 among 233 ICU nurses from intensive care units in multiple tertiary hospitals in China. The survey instruments included a general demographic questionnaire, MD-APPS, PSSS, CD-RISC, and MBI. Descriptive analysis and Pearson correlation analysis were performed using SPSS 27.0. Structural equation modeling was conducted using R 4.4.3 software.

**Results:**

Moral distress was negatively correlated with social support (*r* = −0.765, *p* < 0.05) and psychological resilience (*r* = −0.661, *p* < 0.05), and positively correlated with emotional exhaustion (*r* = 0.714), depersonalization (*r* = 0.737), and reduced personal accomplishment (*r* = 0.706, all *p* < 0.05). The structural equation model demonstrated good fit (*χ*^2^/df = 2.446, CFI = 0.972, TLI = 0.960, RMSEA = 0.079). Moral distress had a significant direct effect on occupational burnout (*β* = 0.380, *p* < 0.05). Social support independently mediated this relationship (*β* = 0.573, 95% CI: 0.320–0.827), accounting for 42.82% of the total effect. Psychological resilience independently mediated the relationship (*β* = 0.174, 95% CI: 0.013–0.335), accounting for 13.01% of the total effect. The chain mediating effect of social support and psychological resilience was significant (*β* = 0.211, 95% CI: 0.055–0.368), accounting for 15.79% of the total effect. The total indirect effect accounted for 71.62% of the total effect.

**Conclusion:**

Social support and psychological resilience play both independent and chain-mediated roles in the relationship between moral distress and occupational burnout among ICU nurses. Healthcare managers should prioritize addressing moral distress and establishing comprehensive support systems to enhance social support and psychological resilience, thereby reducing occupational burnout.

## Introduction

In today’s healthcare system, nurses serve as an indispensable group whose professional well-being directly impacts the quality of medical services and patients’ recovery experience (1). However, in recent years, the issue of occupational burnout among nurses has become increasingly prominent (2), emerging as a significant factor affecting the stability of the nursing workforce and the development of the healthcare industry (3). Occupational burnout is a psychological state characterized by emotional exhaustion, depersonalization, and reduced personal accomplishment, arising from prolonged work stress that remains unaddressed (4). Nurse burnout not only severely compromises their own health, leading to anxiety, depression, and crises of professional identity, but also has profound negative impacts on patient safety and hospital operations. For patients, burnout directly leads to increased rates of medical errors, diminished quality of care, communication gaps, and reduced satisfaction, thereby jeopardizing patient safety. For hospitals, it leads to high turnover rates, incurs substantial labor costs, reduces work efficiency and team morale, damages the hospital’s reputation, and increases the risk of medical incidents and legal liabilities (5). A survey of 155 ICU nurses in Latvia revealed that approximately one-quarter of nurses were considering leaving their jobs. Moral distress showed a significant positive correlation with burnout (*r* = 0.357, *p* < 0.001) and were associated with high workloads and intense emotional stress (6). Research indicates that burnout among Chinese nurses is quite prevalent. A burnout survey conducted on 57,000 nurses revealed that half of them experienced burnout (7). A survey on occupational burnout among nurses in Guizhou and Hunan provinces revealed that the average scores for the emotional exhaustion dimension were 26.58 and 30, respectively. Significant differences were observed in the prevalence rates of depersonalization and reduced personal accomplishment, with uneven distributions across the two regions (8, 9). As a specialized department within the hospital, the ICU concentrates advanced medical equipment and critically ill patients. Its high-intensity, high-risk work environment poses extreme challenges to nurses’ professional competence and psychological resilience (10). ICU nurses not only undertake precise clinical monitoring and emergency resuscitation, but also frequently confront complex moral dilemmas. These include decisions regarding the continuation or discontinuation of life-sustaining treatment, conflicts between patient autonomy and family expectations, and the equitable allocation of resources (11). Such situations often plunge them into moral distress, where “whatever they do may lead to regret” (12). This negative emotional experience stemming from ethical conflicts has been proven to be closely associated with occupational burnout. Prolonged moral distress deplete nurses’ emotional resources, leading to emotional exhaustion, depersonalization, and diminished personal accomplishment, ultimately threatening both the quality of care and nurses’ occupational well-being (13).

**Figure 1:**
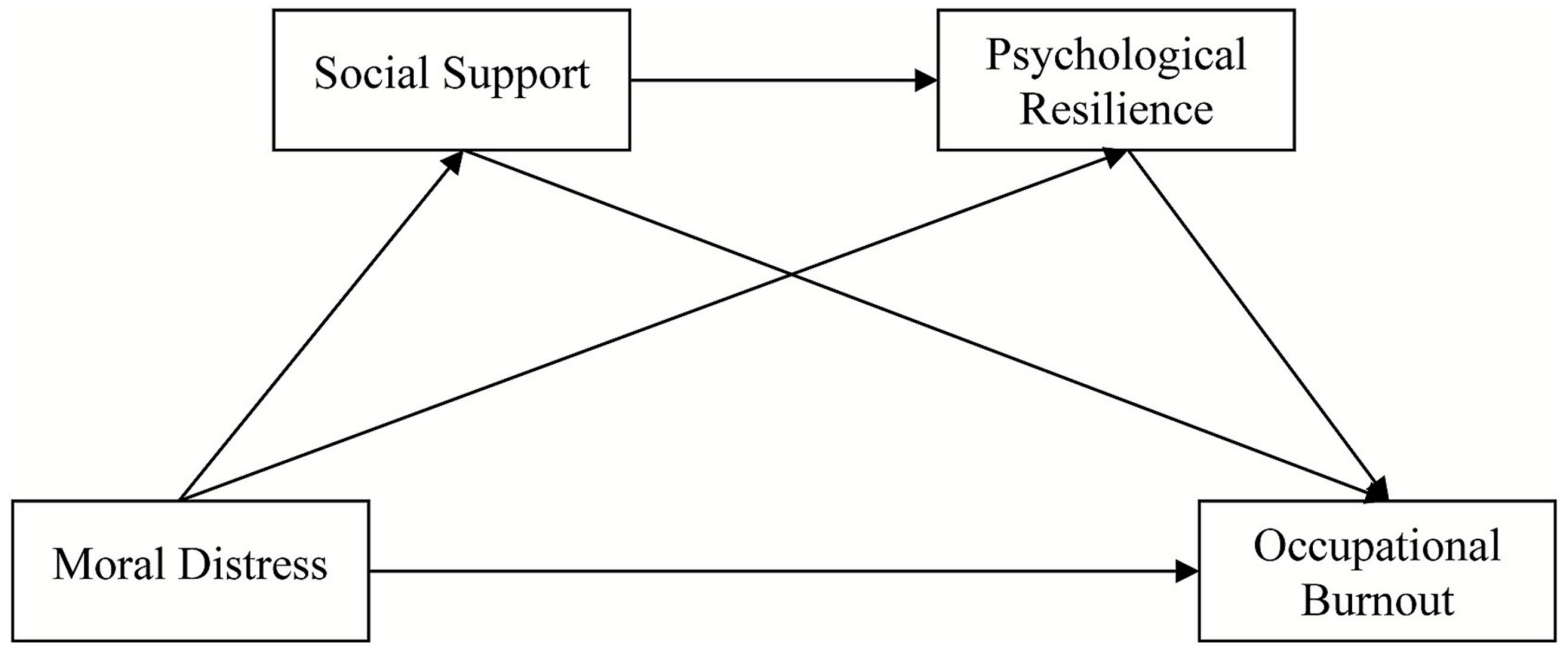
Demonstrated the pathways of mutual influence among moral distress, social support, psychological resilience, and occupational burnout.

Conservation of Resources (COR) theory (14) posits that stress arises from perceived loss or insufficiency of resources. It identifies four resource categories (objective resources, situational resources, personal trait resources, and energy resources), specifically subdivided into internal resources (e.g., psychological resilience, self-confidence, professional skills, emotional regulation ability) and external resources (e.g., medical equipment, organizational support, family relationships, leadership trust). This study reveals the combined effects of external resources (social support) and internal resources (psychological resilience) in the transformation of stressors (moral dilemmas) into negative outcomes (occupational burnout); Social support (15) refers to the aggregate of emotional, material, informational, or practical assistance individuals receive from family, friends, colleagues, communities, and various social organizations in their social lives. Its core function is to help individuals cope with stress, alleviate distress, and maintain mental health and stable social functioning (16). As a crucial external resource for individuals coping with stress, it may serve as a buffer. Social support is also recognized as one of the social factors influencing psychological resilience (17). Wilks et al. (18) found that social support from colleagues, managers, and family serves as a protective factor for psychological resilience. High levels of social support can enhance psychological resilience, promote individuals’ physical and mental health (19), alleviate psychological stress arising from moral distress, and reduce the occurrence of occupational burnout. Psychological resilience is the capacity to recover and “bounce back” from adversity or significant stressors, representing a developable personal trait (20). Kumpfer’s (21) psychological resilience framework indicates that psychological resilience exhibits dynamic changes influenced by both inter as an individual’s inherent resilience, it reflects one’s capacity to adapt and recover in adversity. This resilience may prevent the progression toward burnout by enhancing nurses’ ability to reframe moral distress and mitigating the cumulative effects of negative emotional and external factors (such as family and social environments) (21). Currently, while studies have separately examined the associations between occupational burnout and moral distress, social support, or psychological resilience (22, 23), research systematically integrating these four factors to investigate the mediating role of social support and psychological resilience in the process by which moral distress influence occupational burnout remains relatively scarce. This study contributes to developing strategies for occupational health interventions among ICU nurses. By strengthening social support systems and enhancing psychological resilience, it aims to mitigate the negative effects of moral distress, thereby preventing occupational burnout and stabilizing the ICU nursing workforce. Based on this, this study proposes three model hypotheses (Figure 1): H1: Social support mediates the relationship between moral distress and occupational burnout; H2: Psychological resilience mediates the relationship between social support and occupational burnout; H3: Social support and psychological resilience exhibit chain mediation in the relationship between moral distress and occupational burnout. This study provides scientific evidence for enhancing the professional experience of ICU nurses and optimizing the quality of nursing services.

## Methods

### Research participants

This study employed convenience sampling to conduct a multicenter cross-sectional survey from October to November 2024. A total of 257 ICU nurses from multiple intensive care units across four tertiary hospitals in China were recruited. Prior to data collection, researchers provided all participants with a detailed explanation of the study’s purpose, content, and significance. Participation was voluntary, and participants could withdraw at any stage without consequences. All data were processed anonymously, kept strictly confidential, and used solely for academic research purposes.

Questionnaires were distributed and collected on-site immediately. Two staff members independently cross-checked all responses to ensure data quality. Invalid questionnaires—those completed in an unreasonably short time or showing uniform response patterns—were excluded. Of the 257 questionnaires collected, 233 were deemed valid, yielding an effective response rate of 90.7%.

Inclusion Criteria: (1) Registered nurses currently working in the ICU; (2) Directly involved in critical care; (3) Provided informed consent and volunteered to participate.

Exclusion Criteria: (1) Trainee nurses, rotating nurses, student nurses, or nursing interns; (2) Nurses absent due to vacation, external training, or other reasons.

### Research tools

Collect basic demographic information, including gender, age, department affiliation, educational background, marital status, place of residence, changes in employment status, as well as workload and working hours.

### Moral distress assessment of providers scale (MD-APPS)

The Moral Distress Assessment of Providers Scale (MD-APPS) was originally developed by Belgian scholar Baele et al. (24), designed to assess the severity of moral distress among healthcare providers.

This study employed the Chinese version of the MD-APPS revised by Hu et al. (25). The scale comprises two dimensions and seven items, scored on a 6-point Likert scale ranging from 1 to 6, where “strongly agree” receives 6 points and “strongly disagree” receives 1 point. Items 2, 5, and 7 are positively scored, while the remaining items are reverse-scored. The total score is calculated by summing the scores of all items. A higher total score indicates more severe moral distress experienced by ICU nurses. The Cronbach’s *α* coefficient for this scale in the present study was 0.787.

### Perceived social support scale (PSSS)

This study adapted the Perceived Social Support Scale developed by Huang and Jiang (26), with the primary modification being the replacement of “superiors, relatives, colleagues” with “teachers, classmates, relatives.” The scale comprises 12 items organized into three subscales: family support, friend support, and other support (teachers, classmates, relatives), with each subscale containing 4 items. This scale is a 7-point scale, ranging from 1 = Strongly Disagree to 7 = Strongly Agree. The total social support score is calculated by summing the scores of the three indicators. Higher scores indicate a greater overall level of social support received. The Cronbach’s *α* coefficient for this scale in the present study was 0.969.

### Connor-Davidson resilience scale (CD-RISC)

This study employed the Chinese version of the Connor Davidson Resilience Scale (CD-RISC) revised by Yu and Zhang (20). The questionnaire comprised 25 items rated on a 5-point Likert scale ranging from 0 to 4, denoting “completely disagree,” “rarely agree,” “sometimes agree,” “often agree,” and “completely agree,” respectively. This scale comprises three dimensions: resilience, strength, and optimism. The total score ranges from 0 to 100 points, with higher scores indicating greater psychological resilience. The Cronbach’s *α* coefficient for this scale in the present study was 0.787.

### Maslach burnout inventory (MBI)

The original MBI was developed by Maslach and Jackson (27) to measure levels of burnout. This study employed the Chinese version of the MBI-GS revised by Li et al. (28), comprising three dimensions: Emotional Exhaustion (EE), Depersonalization (DP), and Personal Achievement (PA). The scale consists of 22 items, with the EE dimension measured by 9 items, the DP dimension by 5 items, and the PA dimension by 8 items (reverse-scored). Scoring utilized a 7-point Likert scale, with responses ranging from 0 (“Never”) to 6 (“Daily”). Higher scores on the EE and DP dimensions, along with lower scores on the PA dimension, indicated greater burnout levels. The Cronbach’s *α* coefficient for this scale in the present study was 0.839. This study employs the Chinese version of the Maslach Burnout Inventory—Human Service Survey (MBI-HSS), validated and licensed from Mind Garden, Inc.

### Data analysis

Data analysis was performed using SPSS 27.0 software (IBM SPSS Statistics for Windows, Version 27.0) with the following methods: (1) The Kolmogorov–Smirnov (K-S) one-sample test was used to assess the normality of the data; (2) Descriptive statistical methods were employed to analyze participants’ general information data using SPSS 27.0 software. Pearson’s bivariate correlation analysis was applied to evaluate relationships among key variables including moral distress, social support, psychological resilience, and occupational burnout. (3) Based on the research hypotheses, three structural equation models were constructed using R version 4.4.3. This study employed a bootstrap sampling method with 5,000 samples to examine the chained mediating effects of social support and psychological resilience on moral distress and occupational burnout. Direct and indirect effects were tested using 95% confidence intervals (CI). Various indices were used to evaluate model fit, including the Comparative Fit Index (CFI), Normality of Fit Index (NFI), Tucker-Lewis Index (TLI), Root Mean Square Error of Approximation (RMSEA), Goodness-of-Fit Index (GFI), Chi-Square per Degree of Freedom (*χ*^2^/df), and Standardized Root Mean Square Residual (SRMR). RMSEA ≤ 0.08, CFI, GFI, NFI, TFI > 0.9, *χ*^2^/df < 3.00 (29), indicating acceptable model fit.

## Result

### Demographic characteristics of participants

The baseline demographic characteristics of the 233 participants are shown in Table 1. The majority (125 participants, 53.6%) were aged 20–30 years; 42 participants (18.0%) were male, while 191 (82.0%) were female, indicating a female majority; marital status: 130 participants (55.8%) were married, and 101 (43.3%) were unmarried; educational attainment: 156 participants (67.0%) held a bachelor’s degree, representing the largest group; contract workers constituted the majority (78.6%) among all participants; Most participants rated their rest periods and job satisfaction as average; Most participants (48.1%) rated their rest periods and job satisfaction as average; Most participants believed work hours generally did not impact personal life or family arrangements; The majority reported 16–30 min of seated rest per shift; Annual leave days were mostly 0–5 days; Monthly rest days were mostly 5–8 days.

**Table 1:** Demographic characteristics of participants.

Variables	Nurses (*n* = 233)	
Age		
20-30 year	125	(53.6%)
31-40 year	99	(42.5%)
≥41 year	9	(3.9%)
Gender		
Male	42	(18.0%)
Female	191	(82.0%)
Marital_status		
Unmarried	101	(43.3%)
Married	130	(55.8%)
Cohabiting	1	(0.4%)
Divorced	1	(0.4%)
Education		
Secondary–specialized school	5	(2.1%)
Junior college	66	(28.3%)
Undergraduate	156	(67.0%)
Master’s degree	6	(2.6%)
Nursing_degree		
Junior - college nursing	71	(30.5%)
Undergraduate nursing	157	(67.4%)
Master of Nursing	5	(2.1%)
Monthly_income		
Below 3,000 yuan	11	(4.7%)
3,001–5,000 yuan	71	(30.5%)
5,001–8,000 yuan	107	(45.9%)
8,001–12,000 yuan	43	(18.5%)
12,001–15,000 yuan	1	(0.4%)
Position		
General nurse	141	(60.5%)
Primary nurse	85	(36.5%)
Head nurse	7	(3.0%)
Employment_type		
Full–time	47	(20.6%)
Part–time	1	(0.4%)
Contract worker	184	(78.6%)
Intern nurse	1	(0.4%)
Rest_time_adequacy		
Very sufficient	18	(10.1%)
Relatively sufficient	71	(29.2%)
Average	80	(34.3%)
Not very sufficient	51	(21.9%)
Very insufficient	13	(5.8%)
Break_time_per_shift		
0-15 min	14	(7.8%)
16-30 min	112	(47.9%)
31-45 min	43	(17.9%)
46-60 min	50	(21.5%)
>60 min	14	(6.0%)
Schedule_satisfaction_score		
Extremely dissatisfied	4	(1.7%)
Relatively dissatisfied	18	(7.7%)
Average	112	(48.1%)
Relatively satisfied	56	(24.0%)
Extremely satisfied	43	(18.5%)
Work_life_balance_impact		
Seriously affect	11	(4.7%)
Greatly affect	75	(32.2%)
Generally	112	(48.1%)
Slightly affect	26	(11.2%)
No affect	9	(3.9%)
Annual_leave_days		
0-5 day	142	(61.9%)
6-10 day	77	(32.3%)
11-15 day	13	(5.6%)
>15 day	1	(0.4%)
Monthly_rest_days		
0-4 day	23	(9.7%)
5-8 day	167	(70.4%)
9-12 day	30	(13.6%)
13-16 day	12	(5.8%)
>17 day	1	(0.4%)

### Multicollinearity test

The collinearity test revealed variance inflation factors of 2.720, 2.269, and 3.055 for moral distress, social support, and psychological resilience, respectively, all below the threshold of 5. Therefore, multicollinearity may not affect our estimates.

### A study on the correlation between occupational burnout and moral distress, social support, and psychological resilience

The correlation analysis revealed that ICU nurses’ moral dilemmas showed a significant negative correlation with social support (*r* = −0.765, *p* < 0.05) and psychological resilience (*r* = −0.661, *p* < 0.05), (*p* < 0.05), but showed significant positive correlations with EE, DP, and PA (*r* = 0.714, *r* = 0.737, *r* = 0.706, *p* < 0.05). Social support showed a significant positive correlation with psychological resilience (*r* = 0.726, *p* < 0.05) and a significant negative correlation with EE, DP, and PA (*r* = −0.786, *r* = −0.776, *r* = −0.823, *p* < 0.05) (*p* < 0.05). Psychological resilience showed significant negative correlations with EE, DP, and PA (*r* = −0.743, *r* = −0.672, *r* = −0.677, *p* < 0.05). In this context, Psychological Resilience requires reverse interpretation: higher moral distress scores correlate positively with lower personal accomplishment, and more severe occupational burnout. Lower scores on social support and psychological resilience were associated with lower personal accomplishment (higher scores) and more severe occupational burnout. Detailed data can be found in Table 2.

**Table 2:** Correlation analysis of research variables.

Variable	Moral dilemmas	Social support	Psychological resilience	EE	DP	PA
Moral dilemmas	1					
Social support	−0.765**	1				
Psychological resilience	−0.661**	0.726**	1			
EE	0.714**	−0.786**	−0.743**	1		
DP	0.737**	−0.776**	−0.672**	0.765**	1	
PA	0.706**	−0.823**	−0.677**	0.704**	0.728**	1

EE, emotional exhaustion; DP, depersonalization; PA, personal achievement. **Statistically significant difference (*p* < 0.01).

### Testing the chain-of-intermediaries model

**Figure 2:**
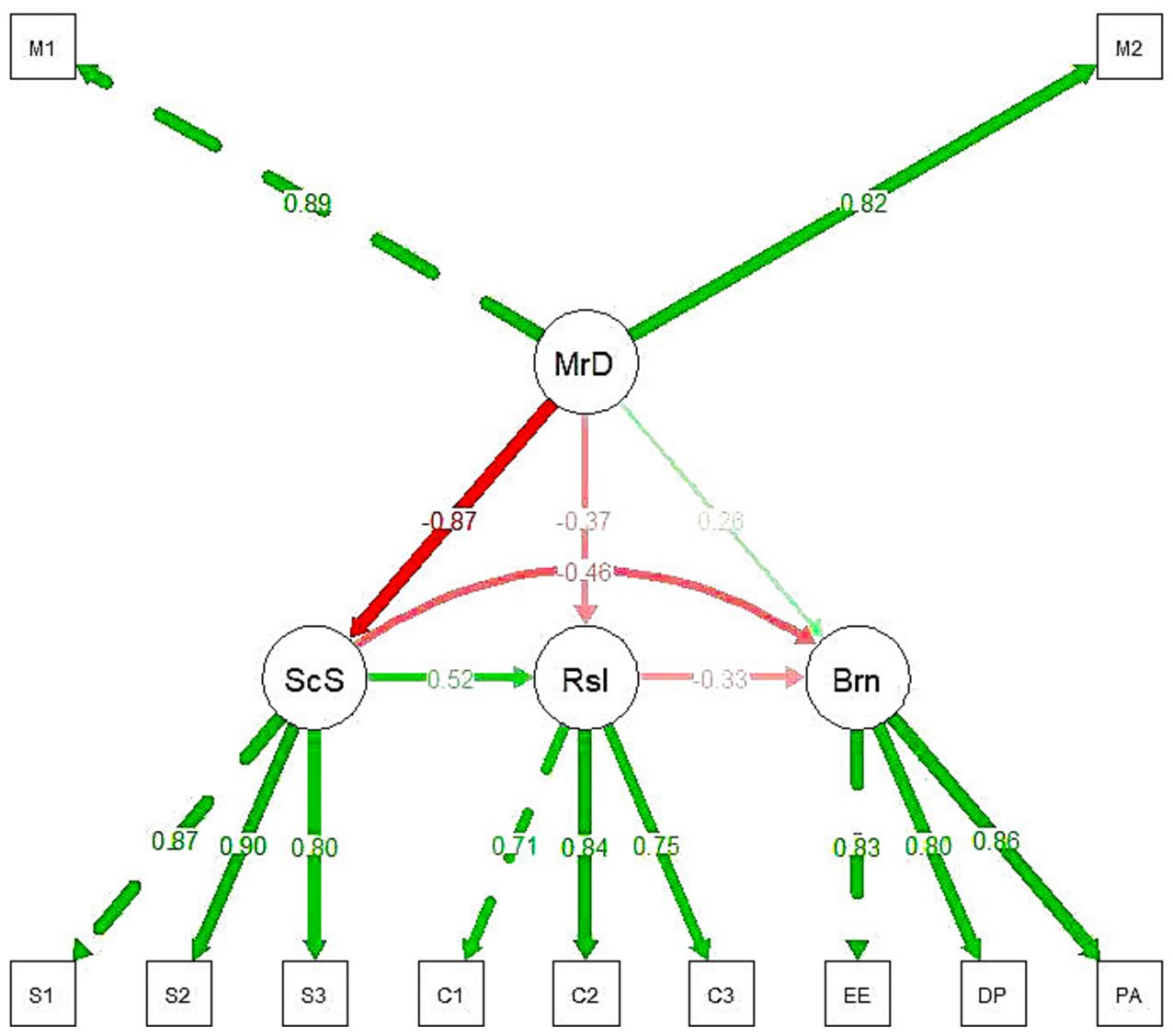
The structural equation model demonstrates the multiple effects of the independent variable moral distress on the dependent variable occupational burnout through the mediating variables social support and psychological resilience, as well as through a direct effect. The magnitude of each path coefficient reflects the strength of the influence, while the sign indicates the direction of the effect.

The chain mediation model was tested using R 4.4.3 software. The structural equation modeling results demonstrated that the model fit indices met the established criteria (*χ*^2^/df = 2.446, GFI = 0.928, NFI = 0.955, TLI = 0.960, CFI = 0.972, SRMR = 0.032, RMSEA = 0.079), indicating that the chain mediation model was valid. The path analysis revealed the following significant relationships: moral distress was negatively associated with social support (*β* = −0.871, *p* < 0.05) and psychological resilience (*β* = −0.371, *p* < 0.05), indicating that higher levels of moral distress were associated with lower social support and reduced psychological resilience; social support was positively associated with psychological resilience (*β* = 0.517, *p* < 0.05), suggesting that greater social support enhanced psychological resilience; moral distress had a significant positive direct effect on occupational burnout (*β* = 0.265, *p* < 0.05); furthermore, both social support (*β* = −0.459, *p* < 0.05) and psychological resilience (*β* = −0.327, *p* < 0.05) were negatively associated with occupational burnout, indicating their protective roles against occupational burnout (Figure 2). These findings support all three hypotheses: (1) moral distress directly influences occupational burnout; (2) social support and psychological resilience independently mediate the relationship between moral distress and occupational burnout; (3) social support and psychological resilience exert a chain mediating effect in this relationship.

### Bootstrap test for mediating effects

A Bootstrap test was conducted using 5,000 bias-corrected Bootstrap samples to assess the statistical significance of the indirect effects. Social support mediated (*β* = 0.573, 95% CI: 0.320–0.827), accounting for 42.82% of the total effect, while psychological resilience mediated (*β* = 0.174, 95% CI: 0.013–0.335), accounting for 13.01% of the total effect. Social support mediated the relationship with psychological resilience (*β* = 0.211, 95% CI: 0.055–0.368), accounting for 15.79% of the total effect. The total indirect effect is 0.959, accounting for 71.62% of the total effect. Detailed data can be found in Table 3.

**Table 3:** Bootstrap analysis of the mediating model.

Paths	*β*	Boot SE	Bootstrap 95% CI (bias corrected)		*p*	Proportion of the total effect (%)
			Lower	Upper		
MD → SS → JB	0.573	0.129	0.32	0.827	0.000	42.82%
MD → PR → JB	0.174	0.082	0.013	0.335	0.034	13.01%
MD → SS → PR → JB	0.211	0.08	0.055	0.368	0.008	15.79%
Indirect effect	0.959	0.133	0.698	0.959	0.133	71.62%
Total effects	1.339	0.096	1.151	1.527	0.000	100.00%

*β*, regression coefficient; SE, standard error; CI, confidence interval.

## Discussion

This study focuses on the specialized group of ICU nurses, investigating the prevalence of occupational burnout among them. It employs a chain mediation model to delve into the intricate relationships among, social support, psychological resilience, and occupational burnout. Previous studies have demonstrated that moral dilemmas directly influence occupational burnout (30). This study, grounded in resource conservation theory, aims to examine the mediating roles and chain mediating effects of social support and psychological resilience among the research variables. It proposes the following core hypotheses: H1: Social support mediates the relationship between moral distress and occupational burnout; H2: Psychological resilience mediates the relationship between moral distress and occupational burnout; H3: A chain mediation effect exists between social support, psychological resilience, and occupational burnout. Structural equation modeling and mediation analysis revealed that H1 was fully supported (social support accounted for 42.82% of the mediating effect, *p* < 0.05); H2 is partially supported (psychological resilience’s sole mediating effect accounts for 13.01%, *p* < 0.05); H3 is fully supported (chained mediating effect accounts for 15.79%, *p* < 0.05). This research, by integrating the COR theory, reveals how external (social support) and internal (psychological resilience) resources buffer the impact of moral distress on occupational burnout. The findings of this study not only reveal the factors influencing occupational burnout but also provide actionable interventions, such as offering social support and training to enhance psychological resilience, which can reduce the incidence of burnout among nurses.

### The relationship between moral distress and occupational burnout among ICU nurses

The study findings clearly demonstrate that ICU nurses’ moral distress exhibit strong positive correlations with all dimensions of occupational burnout (EE, DP, PA), with correlation coefficients ranging from 0.706 to 0.737 (*p* < 0.05). This aligns with the core tenets of Conservation of Resources (COR) theory. This finding is supported by numerous studies. For instance, research conducted by Wu et al. (13) revealed that ICU nurses’ ethical dilemmas showed significant positive correlations with EE (*r* = 0.573, *p* < 0.001) and DP (*r* = 0.510, *p* < 0.001) in the Moral Burnout Inventory (MBI) (*p* < 0.001) and DP (*r* = 0.510, *p* < 0.001). The study reported an average moral distress score of 51.05 ± 15.86 and an average burnout score of 61.86 ± 24.19, further validating that moral distress serve as a key precursor to occupational burnout in this professional group. Research by Salas-Bergüés et al. indicates that moral distress can reduce the occurrence of occupational burnout through organizational and interpersonal relationships (31), further demonstrating that alleviating moral distress is the primary breakthrough for mitigating burnout among ICU nurses. This supports our research findings. From a clinical perspective, ICU nurses face daily moral distress such as “patient selection under resource constraints” and “balancing quality of life with survival time.” For instance, when medical resources are scarce, nurses must allocate ventilators among multiple critically ill patients. This “forced choice” induces moral distress that continuously depletes their emotional reserves. Initially manifesting as anxiety and irritability (EE) during shifts, it progresses to indifference toward patient needs (DP), ultimately undermining their professional self-worth (PA).

This not only severely erodes nurses’ physical and mental well- being, but also poses a direct and serious threat to nursing quality and patient safety. Therefore, deeply understanding and effectively addressing moral distress has become crucial to alleviating nurse burnout and enhancing the quality of care.

### The mediating role of social support

The findings of this study indicate that social support partially mediates the relationship between moral distress and occupational burnout, with a mediation effect value of *β* = 0.573 (95% confidence interval: 0.320–0.827), accounting for 42.82% of the total effect. Social support is negatively correlated with emotional exhaustion and dehumanization dimensions, while positively correlated with personal accomplishment, consistent with Weigel (32) findings. This indicates that enhancing social support can effectively mitigate the negative impact of moral distress on occupational burnout, aligning with the “resource replenishment” mechanism proposed by COR theory. Research indicates that managerial support (such as flexible scheduling and ethical consultation channels) and peer support (such as sharing experiences in ethical decision-making) can reduce burnout rates among ICU nurses by 41%. Nurses lacking social support are 2.3 times more likely to experience burnout stemming from moral distress compared to those receiving support (33). Cao et al. (34) surveyed 244 nurses and found that higher levels of social support, across all dimensions, were negatively correlated with emotional exhaustion and depersonalization dimensions of burnout (*r* = −0.438, *p* < 0.01; *r* = −0.372, *p* < 0.01), and positively correlated with personal accomplishment (*r* = 0.291, *p* < 0.01). For ICU nurses, social support from colleagues, managers, and family members provides emotional comfort, practical assistance, and guidance in ethical decision-making (35, 36). For instance, when nurses encounter moral distress, timely communication with colleagues possessing extensive experience in ethical decision-making can help resolve cognitive confusion. Managers implementing flexible scheduling and providing ethical consultation channels can alleviate the stress arising from role conflicts and moral distress. Social support, as a vital external resource for individuals coping with stress, provides ICU nurses with warmth and strength, effectively alleviating the psychological pressure arising from their moral distress. This external support is crucial, as it reduces the excessive depletion of nurses’ emotional resources, thereby lowering the risk of occupational burnout (37, 38). The study profoundly underscores the critical importance of organizational and societal support systems in safeguarding nurses’ occupational health. Hospital administrators must fully recognize this imperative and actively establish comprehensive, multi-tiered social support networks. These networks should encompass providing professional psychological counseling services, organizing diverse team-building activities, and strengthening communication and interaction between superiors and subordinates. Such measures will effectively assist nurses in navigating the ethical challenges inherent in their work (39, 40).

### The mediating role of psychological resilience

In this study, psychological resilience also demonstrated a significant mediating effect in the relationship between moral distress and occupational burnout, with an effect size of *β* = 0.174 (95% confidence interval: 0.013–0.335), accounting for 13.01% of the total effect. A study by Orgambídez et al. found consistent results with this research, showing that psychological resilience is negatively correlated with occupational burnout [*r* = −0.44, 95% CI (−0.51, −0.36), *n* = 6,092], and also negatively correlated with EE [*r* = −0.32, 95% CI (−0.42, −0.21), *n* = 3,349] (41). A U.S. study indicates that the COVID-19 pandemic has disrupted ICU nurses’ moral distress and mental health, increased the incidence of occupational burnout, and heightened the risk of nurses leaving the ICU profession (42). Research also indicates that nurses’ sense of professional mission enhances psychological resilience, thereby reducing occupational burnout (43). As an internal psychological resource, psychological resilience enables ICU nurses to adjust their cognitive and emotional responses when confronting moral distress. Specifically, nurses with high psychological resilience can more rationally view the inevitability of moral distress in clinical practice, thereby avoiding excessive self-blame and the accumulation of negative emotions, which reduces the risk of professional burnout. They are also better equipped to reframe their perception of moral distress, viewing challenges as opportunities for growth rather than burdensome obligations. This cognitive approach reduces the accumulation of negative emotions. As a predictor of occupational burnout, psychological resilience enables early identification and intervention, thereby blocking the pathway to burnout (43). Therefore, enhancing nurses’ psychological resilience has become an effective approach to preventing occupational burnout. Hospitals can help nurses strengthen their self-regulation abilities by implementing systematic psychological resilience training and offering mindfulness-based stress reduction courses. This enables them to better cope with workplace pressures and challenges, achieving both personal and professional growth.

### Chain mediation effect of social support and psychological resilience

A key innovation of this study lies in the discovery of a chain mediating effect between ICU nurses’ social support and psychological resilience in the relationship between moral distress and occupational burnout. The effect size was *β* = 0.211 (95% confidence interval: 0.055–0.368), accounting for 15.79% of the total effect. This implies that a supportive social environment not only directly alleviates the psychological stress arising from moral distress but also indirectly reduces the occurrence of occupational burnout by enhancing nurses’ psychological resilience (37). This study finds that social support (42.82%), psychological resilience (13.01%) and their chain mediation (15.79%) show hierarchical transmission: social support acts as the core mediating mechanism, psychological resilience plays an auxiliary role, and the chain effect supplements the indirect interaction logic. With the total mediating effect exceeding 70%, indirect effects dominate the variable relationship, and interventions should prioritize social support followed by psychological resilience cultivation. This study demonstrates that by simultaneously strengthening external support systems and enhancing internal resilience, we can more effectively improve the professional experience of ICU nurses, increase their job satisfaction and the quality of care they provide, thereby stabilizing the nursing workforce and elevating the overall standard of healthcare services.

Our research holds significant practical implications for enhancing the professional experience of ICU nurses and improving the quality of nursing services. The findings provide clear guidance for occupational health interventions: by strengthening social support systems—such as establishing peer support groups and implementing family care policies—and enhancing nurses’ psychological resilience through resilience training and counseling hotlines, the negative impact of moral distress on ICU nurses can be effectively mitigated, thereby preventing the onset of occupational burnout. More importantly, reducing job burnout helps improve ICU nurses’ job satisfaction and retention rates, thereby stabilizing the nursing workforce and ensuring the continuity and high-quality development of healthcare services. Finally, enhancing the professional experience of ICU nurses can also inspire their enthusiasm and creativity, elevate the professionalism and humanistic care of nursing services, and thereby optimize patients’ recovery experience and satisfaction. In summary, our research not only deepens our understanding of the relationship between moral distress and occupational burnout among ICU nurses, but also provides valuable scientific evidence and practical guidance for future occupational health management and improvements in nursing service quality.

### Limitations and future development directions

Although our research has yielded certain findings, there remain some limitations. Regarding sample representativeness, the use of convenience sampling—selecting only four tertiary hospitals in Liaoning and Jiangxi provinces—may result in the sample failing to comprehensively reflect the overall situation of ICU nurses nationwide. In terms of research design, the cross-sectional survey approach precludes establishing causal relationships among moral distress, social support, psychological resilience, and occupational burnout.

Furthermore, research relying on self-reported data from nurses may be subject to information bias or social desirability bias, which could compromise the accuracy of the findings. Regarding the limitations of this study, future research may be conducted in the following areas: Expand the sample scope to include hospitals across different regions and tiers to enhance the study’s external validity. Employing a longitudinal design to track changes over time in variables such as moral distress, social support, psychological resilience, and occupational burnout, this approach more accurately reveals the dynamic causal relationships among them. By integrating objective metrics such as nursing quality indicators and patient satisfaction into a comprehensive assessment, we address the limitations of self-reported data, thereby enhancing the reliability and accuracy of research findings.

## Conclusion

This study found that ICU nurses’ moral distress were significantly positively correlated with occupational burnout. Moral distress directly influence nurses’ occupational burnout and indirectly affect it through social support and psychological resilience. This study found that social support and psychological resilience partially mediate the relationship between moral distress and occupational burnout, jointly forming a chained mediating effect. This highlights the importance of enhancing ICU nurses’ levels of social support and psychological resilience. This study holds significant implications for ICU nursing practice. It provides clear directions for occupational health interventions for nurses—such as strengthening social support and enhancing psychological resilience to prevent burnout—thereby helping stabilize the nursing workforce, improve nursing quality, and enhance patient recovery experiences. Furthermore, it offers scientific evidence for hospital management, facilitating the development of more comprehensive strategies to safeguard nurses’ occupational health and elevate the quality of hospital medical services.

## Data Availability

The original contributions presented in the study are included in the article/supplementary material, further inquiries can be directed to the corresponding author.
